# An epidemiological survey of *Dirofilaria* spp. and *Acanthocheilonema* spp. in dogs from the Republic of Moldova

**DOI:** 10.1186/s13071-021-04891-3

**Published:** 2021-08-06

**Authors:** Mirabela Oana Dumitrache, Gianluca D’Amico, Eugeniu Voiniţchi, Serghei Maximenco, Viorica Mircean, Angela Monica Ionică

**Affiliations:** 1 Department of Parasitology and Parasitic Diseases, University of Agricultural Sciences and Veterinary Medicine Cluj-Napoca, 3-5 Mănăştur Street, 400372 Cluj-Napoca, Romania; 2 Faculty of Veterinary Medicine, State Agrarian University of Moldova, 48 Mircești Street, Chişinău, Republic of Moldova; 3 CDS-9, “Regele Mihai I Al României” Life Science Institute, University of Agricultural Sciences and Veterinary Medicine Cluj-Napoca, 3-5 Mănăştur Street, 400372 Cluj-Napoca, Romania; 4 Agenţia Naţională pentru Siguranţa Alimentelor, MD3900 str. Griviţei, 28, Cahul, Republic of Moldova

**Keywords:** *Dirofilaria*, *Acanthocheilonema*, Vector-borne disease, Zoonosis, Dog, Republic of Moldova

## Abstract

**Background:**

During the last decades, filarial infections caused by *Dirofilaria* spp. have spread rapidly within dog populations of several European countries. Increasing scientific interest in filariasis, and the availability of new diagnostic tools, has led to improved knowledge of the biology, morphology, and epidemiology of different species of filarial worms. However, data are still scarce for a number of countries, including the Republic of Moldova. Thus, we assessed the epidemiological status of canine filariasis in the Republic of Moldova to address part of this knowledge gap.

**Methods:**

A total of 120 blood samples were collected between June 2018 and July 2019 from dogs originating from the cities of Cahul and Chişinău. The samples were examined microscopically, and multiplex polymerase chain reaction was performed to evaluate filarioid species diversity.

**Results:**

Microscopic examination revealed that 12 dogs (10.0%) were positive for circulating microfilariae. The molecular test showed that one dog was positive for *Acanthocheilonema reconditum* (0.8%), one for *Dirofilaria* *immitis* (0.8%), six for *Dirofilaria* *repens* (5.0%), and four (3.3%) harboured a co-infection with *D*. *immitis* and *D*. *repens*. Prevalence was significantly higher in dogs aged ≥ 2 years.

**Conclusions:**

The epidemiological survey presented here for the Republic of Moldova confirmed the presence *D*. *immitis*, *D*. *repens* and *A*. *reconditum* in dogs that had not received any heartworm preventive.

**Graphical abstract:**

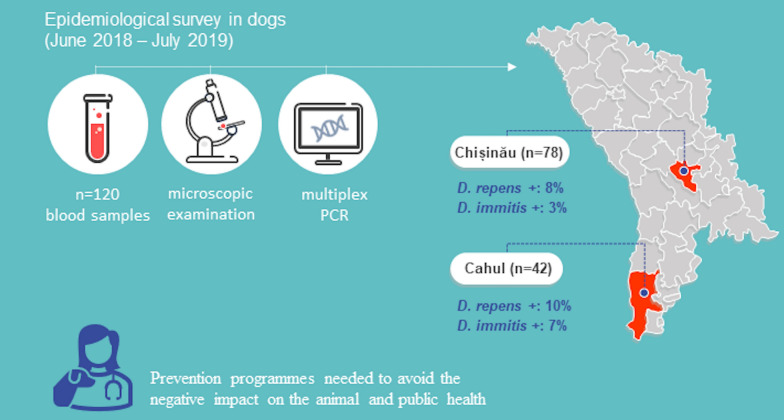

## Background

Filarioids (superfamily Filarioidea) are vector-borne nematodes that pose a health risk to both domestic and wild animals and to humans [1]. Numerous filarial species have been identified and characterized by morphological and molecular methods during the last decades [2–4]. Moreover, the evolution of existing diagnostic tools and the availability of new ones have enabled an increase in our knowledge of the epidemiology and ecology of many filarial species [1]. Although some species have been intensively studied, and awareness of filarioid infections for various mammals is high (e.g. infections with *Dirofilaria immitis* and *Dirofilaria* *repens*), other filarial species have been rather neglected (e.g. *Cercopithifilaria bainae* and *Cercopithifilaria* *grassii*) or are less well known (e.g. *Acanthocheilonema reconditum*, *Acanthocheilonema* *dracunculoides* and *Onchocerca lupi*) [4].

Dirofilariasis is one of the most studied and best-known parasitic diseases. It is caused by mosquito-borne nematodes, of which *D*. *immitis* and *D*. *repens* are the most important [4, 5] due to their high pathogenicity, potential negative impact on public health, wide distribution and endemicity [5]. Both *D*. *immitis* and *D*. *repens* can be transmitted by several genera of culicid vector (*Anopheles*, *Aedes*, *Ochlerotatus*, *Culex*, *Culiseta* and *Coquillettidia*) [6]. *D. immitis* may cause a severe cardiopulmonary condition in dogs and other domestic, and wild, carnivores [5]. Although humans are considered to be accidental hosts, infections occasionally occur and can cause pulmonary conditions and, in some instances, ocular or subcutaneous diseases [7–9]. *D. repens* is the agent of subcutaneous dirofilariasis in animals, which usually has a mild clinical manifestation or is unrecognised. However, various skin lesions may occur, such as non-inflammatory nodules, circular alopecia, localised erythema, and lichenification and hyperpigmentation of affected areas in chronic cases. Depending on their localization and immune reactions, these lesions may be pruritic or painful, but are usually neither [10]. *D. repens* is the main agent of dirofilariasis in humans, in whom it most frequently causes ocular and subcutaneous diseases. Other localisations (e.g. pulmonary, oral cavity, eyelid) of the disease have also been reported [9]. In Europe, *D. repens* is recognised as an emergent pathogen. Microfilariaemic dogs represent the main reservoir for animal and human infections with *D*. *immitis* and *D*. *repens *[10]. A northeastern multifactorial spread (due to influences such as climate change, vector availability, dog and human circulation, etc.) of both *D*. *immitis* and *D*. *repens* in areas previously considered non-endemic for dirofilariasis has been recently observed [4]. These two species, as well as *Thelazia callipaeda*, another vector-borne pathogen of dogs, are considered key examples for this pattern of emerging parasitic disease [4]. Thus, epidemiological studies in areas where no or limited information is available on them, but where they are expected to be present, are essential.

Another filarioid species that parasitises dogs, and has been reported once in a human patient [11], is *A*. *reconditum*. Although this species is the most widely spread filarial worm and has a global distribution, it is one of the less pathogenic filarioids in dogs, and its low clinical impact has been previously demonstrated [4, 12]. However, this parasite, which is mainly found beneath the subcutaneous tissues of the limbs and dorsal region of dogs, might be responsible for alopecia and/or dermatitis in the same areas [4, 12]. The life cycle of *A*. *reconditum*, unlike that of any other filarioid, depends on several species of fleas (*Ctenocephalides canis*, *Ctenocephalides felis*, *Pulex irritans*, *Pulex* *simulans*, and *Echidnophaga gallinacea*) or lice (*Linognathus setosus* and *Heterodoxus spiniger*), which serve as vectors and intermediate hosts [4].

Several methods are used for the diagnosis of filarial infections, such as microscopic detection of circulating microfilariae, a commercial test designed to detect the presence of blood antigens released by adult females of *D*. *immitis*, and molecular-based methods such as polymerase change reaction (PCR) and duplex real-time PCR [6, 13]. Echocardiography is also used for the diagnosis of *D*. *immitis* in dogs [4].

Even though the veterinary significance of *D*. *immitis*, *D*. *repens* and *A*. *reconditum* has been acknowledged [4, 5], there are still some gaps in knowledge regarding their epidemiology and biology. Two recent epidemiological studies suggested the presence and the circulation of *Dirofilaria* spp. in humans [14] and in arthropod vectors [15] in the Republic of Moldova, but a correlation analysis between the prevalence and geographical distribution of these nematodes in canine and human populations could not be carried out due to the lack of epidemiological studies on dogs. To address this lack, our study assessed the prevalence of canine filariasis in dog populations in the Republic of Moldova and the associated risk factors.

## Methods

### Sampling

Convenience sampling of 120 dogs was performed between June 2018 and July 2019. The dogs originated from public dog shelters located in the south (Cahul, *n* = 42) and central (Chişinău, *n* = 48) parts of the country, and from veterinary clinics in Chişinău (*n* = 30). All the shelter dogs were housed outdoors, while the owned dogs had a mixed lifestyle. Only owned animals that did not receive any kind of preventive treatment for dirofilariasis were included in the study. We assumed that the dogs from the shelters had not received any kind of chemoprophylactic treatment against filarial infections. Informed consent was obtained from the dog owners and managers of the shelters before the inclusion of the dogs in the study.

Blood samples were collected from the cephalic vein of each dog (2 ml) and stored in a labelled tube with anticoagulant (ethylenediaminetetraacetic acid). Location, sex, age, breed, origin and travel history were recorded for each dog, to assess the risk factors for *Dirofilaria* spp. and *A*. *reconditum* infections.

### Microscopic examination

For each dog, 1 ml of blood was processed by the modified Knott’s test according to the standard procedure [16]. The sediments were examined using an Olympus BX61 microscope. Microfilariae were identified based on their morphology [16]. Photographs and measurements for morphological identification were obtained using a DP72 camera and Cell^F software (Olympus, Tokyo, Japan).

### Molecular analysis

Genomic DNA was extracted from 200 μl of whole blood using a commercial kit (Isolate II Genomic DNA Kit; Bioline, London) according to the manufacturer’s instructions. Multiplex PCRs amplifying partial regions of the cytochrome c oxidase subunit 1 gene of three filarioid species [*D*. *immitis*, 169 base pairs (bp); *D*. *repens*, 479 bp; and *A*. *reconditum*, 589 bp] were performed using species-specific forward primers and the reverse primer NTR, as described in the literature [17]. The PCR products were visualized by electrophoresis in a 2% agarose gel stained with RedSafe 20,000× Nucleic Acid Staining Solution (Chembio, Hertfordshire, UK), and their molecular weights determined by comparison to a molecular marker (HyperLadder 100bp; Bioline). All the corresponding bands were excised from the gel and purified using a commercially available kit (Isolate II PCR and Gel Kit; Bioline). The purified products were sequenced by an external service (Macrogen Europe, Amsterdam). The obtained sequences were compared to those available in GenBank by a Basic Local Alignment Search Tool (BLAST) analysis.

### Statistical analysis

Data analysis was performed using Epi Info 7 software (Centers for Disease Control and Prevention, USA). The frequency and prevalence of infection are reported with 95% confidence intervals (CIs), and the risk factors (locations, sex, age, breed, and origin of the animals) were assessed using a Chi-square test. Differences were considered statistically significant at *P* < 0.05.

## Results

Microscopic examination revealed that 12 dogs (10.0%; 95% CI 5.3–16.8) were positive for circulating microfilariae. Age had a significant effect on filarioid infection, as all of these dogs were ≥ 2 years old (Chi-square test, χ^2^ = 2.91, *df* = 1, *P* = 0.038). There were no significant differences regarding location, sex, breed or origin of the animals (Table 1).

**Table 1:** Prevalence of filarioid infections (regardless of species) in sampled dogs from the Republic of Moldova

Variables	Frequency	Prevalence	95% CI	χ^2^ (*df*)	*P*-value
Location					
Chișinău	7/78	9.0%	3.7–17.6	0.036 (1)	0.751
Cahul	5/42	11.9%	4.0–25.6		
Sex					
Male	2/50	4.0%	0.5–13.2	2.381 (1)	0.072
Female	10/70	14.3%	7.1–24.7		
Age					
< 2 Years	0/29	0.0%	0.0–11.9	2.91 (1)	0.038
≥ 2 Years	12/91	13.2%	7–2.9		
Breed					
Pure breed	1/11	9.1%	0.2–41.3	0 (1)	1
Mixed breed	11/109	10.1%	5.2–17.3		
Origin					
Shelter	11/90	12.2%	6.3–20.8	1.111 (1)	0.290
Owned	1/30	3.3%	0.1–17.2		
Total	12/120	10.0%	5.3–16.8	–	–

*CI* Confidence interval

Among the microfilariaemic dogs, one was positive for *Acanthocheilonema* sp. (0.8%; 95% CI 0.0–4. 6), one for *D*. *immitis* (0.8%; 95% CI 0.0–4.6), six for *D*. *repens* (5.0%; 95% CI 1.9–10.6), and four (3.3%; 95% CI 0.9–8.3) harboured a co-infection with *D*. *immitis* and *D*. *repens*. Infection with *Acanthocheilonema* spp. was identified only in Cahul, while *Dirofilaria* spp. were detected at both locations. Although prevalence tended to be higher for both *Dirofilaria* spp. in Cahul than in Chişinău, the difference was not statistically significant. No other significant risk factors were identified (Tables 2, 3).

**Table 2:** Prevalence of *Dirofilaria* *repens* infection in sampled dogs from the Republic of Moldova

Variables	Frequency	Prevalence	95% CI	χ^2^ (*df*)	*P*-value
Location					
Chișinău	6/78	7.7%	2.9–16.0	0 (1)	1
Cahul	4/42	9.5%	2.7–22.6		
Sex					
Male	2/50	4.0%	0.5–13.2	1.246 (1)	0.19
Female	8/70	11.4%	7.1–24.7		
Age					
< 2 Years	0/29	0.0%	0.0–12.0	2.186 (1)	0.115
≥ 2 Years	10/91	11.0%	5.2–19.3		
Breed					
Pure breed	1/11	9.1%	0.2–41.3	0 (1)	1
Mixed breed	9/109	8.3%	3.9–15.1		
Origin					
Shelter	9/90	10.0%	4.7–18.1	0.581 (1)	0.448
Owned	1/30	3.3%	0.1–17.2		
Total	10/120	8.3%	4.1–14.8	–	–

**Table 3:** Prevalence of *Dirofilaria* *immitis* infection in sampled dogs from the Republic of Moldova

Variables	Frequency	Prevalence	95% CI	χ^2^ (*df*)	*P*-value
Location					
Chișinău	2/78	2.6%	0.3–9.00	0.516 (1)	0.341
Cahul	3/42	7.1%	1.5–19.5		
Sex					
Male	2/50	4.0%	0.5–13.7	0 (1)	1
Female	3/70	4.3%	0.9–12.0		
Age					
< 2 Years	0/29	0.0%	0.0–11.9	0.571 (1)	0.334
≥ 2 Years	5/91	5.5%	1.8–12.4		
Breed					
Pure breed	0/11	0.0%	0.0–29.5	0 (1)	1
Mixed breed	5/109	4.6%	1.5–10.4		
Origin					
Shelter	5/90	5.6%	1.8–12.5	0.626 (1)	0.329
Owned	0/30	0.0%	0.0–11.6		
Total	5/120	4.2%	1.4–9.5	–	–

The molecular analysis confirmed the microscopy outcomes. In the case of *Acanthocheilonema* spp. infection, the sample was PCR positive for *A*. *reconditum*, while the BLAST analysis revealed a 100% nucleotide similarity to an *A*. *reconditum* sequence from a dog from Italy (GenBank: JF461456). For *D*. *immitis*, all five sequences were identical, and had 100% nucleotide similarity to other isolates from Europe and Asia (e.g. GenBank LC107816, KF692101, MK250715, KR870344). Two different sequences were obtained from the *D*. *repens* isolates. The first one, identified from nine dogs, was 100% identical to three other European isolates from human cases (GenBank: KR998257, KX265049) and mosquitoes (GenBank: MF695085). The second sequence, identified from a dog originating in Cahul, was 100% similar to an isolate from a human case (GenBank: AB973225), a Japanese woman after she had travelled to Europe.

## Discussion

The Republic of Moldova is one of the countries for which few epidemiological data are available on *Dirofilaria* spp. and *A*. *reconditum*. A lack of diagnostic tools, misdiagnosis, and the low awareness of doctors and veterinarians of dirofilariasis are considered to be the main factors responsible for this gap in knowledge [15]. In a molecular study conducted from 2010 to 2015, of 347 pools of female mosquitoes analysed, 92 and 30 tested positive for *D*. *repens* and *D*. *immitis*, respectively. From their analysis of the geographic distribution and temperatures of the sites where the positive sample were collected, the authors concluded that the entire country has favourable climatic conditions for the transmission of *Dirofilaria* spp. [15]. The results of our study are in line with this conclusion. Only one previous study has reported the presence of *D*. *immitis* in canine populations in the Republic of Moldova. A total of 13 shepherd dogs originating from two counties, Ialoveni and Criuleni, located in the central part of Moldova, were evaluated for the presence of various parasite species by necropsy, and three of the examined dogs were found to be infected with *D*. *immitis* [18]. The method used to identify the parasites was not given, and it was also unclear if the positive dogs originated from the same county. Although both *D*. *immitis* and *D*. *repens* have been found in the human population of the Republic of Moldova, most of the available information comprises individual case reports, where the nematodes were identified based on microscopic examination only. Five cases of human dirofilariasis were reported up until 2016 [15]. However, it is not clear if these cases were autochthonous or imported [15]. An extensive study on the human population of the Republic of Moldova was performed in 2018, when 263 serum samples were screened for exposure of individuals to *Dirofilaria* spp. One sample was positive for *D*. *repens* antigens, 36 were positive for anti-*D*. *immitis* immunoglobulin G, and three samples were reactive for antigens of both *D*. *immitis* and *D*. *repens* [14].

Although previous studies found that sex [19] or breed [20] are risk factors for *Dirofilaria* spp. infection in dogs, our study found no significant differences regarding location, sex, breed or origin of the animals. This might be due to the low numbers of samples used. However, prevalence of infection was significantly higher for dogs aged ≥ 2 years. Age was previously highlighted as one of the most important risk factors for infection [20, 21]. Our study included two categories of dogs: those that were owned, and those that lived in a shelter. Considering that, to our knowledge, none of the dogs, regardless of their origin, had received prophylactic treatment for dirofilariasis, we can assume that the difference in prevalence between the two categories, although not statistically significant, was related to the length of time that they spent outside, and consequently, their potential exposure to the vectors.

Infections with *D*. *repens* and *A*. *reconditum* have not been previously reported for the Republic of Moldova. However, in Ukraine, which borders the Republic of Moldova to the east, north and south, and Romania, the western neighbour state, many infections with these pathogens have been reported in human and animal populations. In Ukraine, dirofilariasis caused by *D*. *repens* was first reported in dogs in 1904 and in humans in 1927 [22]. Between 1997 and 2013, a total of 1465 cases of infection with *D*. *repens* were confirmed in humans. The incidence of *Dirofilaria* infection ranged between 0.07–3.71 per 100,000 people in the geographical areas neighbouring the Republic of Moldova. Due to the presence of the pathogen in all the oblasts of Ukraine, as well as the high incidence registered in many regions, the authors concluded that dirofilariasis due to *D*. *repens* is an emergent zoonosis in the country [22]. Rossi et al. [23] demonstrated the implication of both *D*. *immitis* and *D*. *repens* in ocular and subcutaneous pathologies in humans in Ukraine. In Romania, sporadic infections with *D*. *immitis* in dogs had been reported from the beginning of the twentieth century, but more recent studies, which used various diagnostic methods, revealed prevalences ranging from 23.1% to 38.0% [24, 25]. In 2014, both *D*. *immitis* and *D*. *repens* were categorized as endemic in areas of southern and southeastern Romania, and the same study provided the first extensive overview of the prevalence and distribution of *A*. *reconditum* in the country [26]. Taking into consideration the epidemiological situation in Europe, and in particular in the two neighbouring countries of the Republic of Moldova—Romania and Ukraine—as well as the favourable climatic conditions for the transmission of *Dirofilaria* spp. in the former, it is highly probable that the limited number of reports of these pathogens in the Republic of Moldova is the result of a lack of targeted epidemiological studies.

During the last decades, infections with *Dirofilaria* spp. in dogs have spread from the traditionally endemic regions of Italy, Spain, France, [27] into central, eastern and northeastern European countries such as Switzerland [10], Germany [28], Austria [29], Czech Republic [30], Poland [31] and Romania [25]. Interestingly, though, a new trend in the distribution of these pathogens has recently been observed: the prevalence of dirofilariasis has decreased in the last few years in western Europe in areas of high endemicity. This has been attributed to increased awareness of these diseases and, as a consequence, greater acceptance and widespread use of preventative measures [32]. In contrast, in non-endemic regions, or where these parasites have not been previously reported (including eastern European countries), recent data have shown first cases and/or an increase in the prevalence of these helminth infections. The spread of these pathogens is likely facilitated by climate change, the lack of experience of veterinary practitioners in diagnosing and treating infections with them, poor awareness of these diseases amongst both medical personnel and dog owners, and a high number of stray dogs [32]. Austria, which reported the presence of autochthonous *D*. *repens* for the first time in 2012 [33], announced that infections with *D*. *immitis* and *D*. *repens* had tripled by 2018 [34]. Similar epidemiological patterns have been reported for Romania and Ukraine [22, 35].

Many methods have been proposed for the diagnosis of filarioid infections in dogs. However, a lack of sensitivity of microscopic methods for the detection of larvae, cross-reactivity with *Angiostrongylus vasorum* of some commercially available antigen tests for *D*. *immitis*, and false negative results found with the same type of test where the parasitic burden is low, are the main limitations of these diagnostic tools [4, 23]. PCR, duplex real-time PCR and multiplex PCR are considered useful diagnostic tools for both epidemiological and clinical studies [4]. Our study presents the results of epidemiological screening using two types of complementary tests, i.e. microscopic and molecular, that should strengthen the reliability of the presented data.

Dogs are the main reservoirs of *Dirofilaria* spp., and thus serve as a source of infection for mosquito vectors and possible subsequent transmission to humans and other susceptible mammalian hosts [5]. Monitoring the canine population is an important step for the design of prevention programmes aimed to decrease the risk of zoonoses caused by *Dirofilaria* spp. The need for more detailed information and the development of monitoring programmes and epidemiological studies on dirofilariasis and other zoonotic vector-borne pathogens in dogs from the Republic of Moldova has been previously highlighted [14, 15]. More data on the prevalence and geographical distribution of *Dirofilaria* spp. in dogs, humans and vectors would allow a better understanding of the circulation of these pathogens in the Republic of Moldova. Although we recognize that the sample size used here is not high, the results of our study should raise awareness amongst veterinarians and physicians. The circulation of dogs between different countries, with their owners or for commercial reasons, is becoming increasingly common. In light of the current study, dogs originating from the Republic of Moldova should be screened for *Dirofilaria* spp. and *Acanthocheilonema* spp. Moreover, preventive measures are advisable for dogs (and their owners) entering the Republic of Moldova.

The role of stray dogs in the circulation of these filarial worms, and thus the high risk that they pose for human health, has been previously demonstrated [36]. Although origin was not identified as a risk factor in our study, possibly due to the small sample size, we believe that stray dogs could act as an important source of infection with filarial worms in other carnivores and in humans. The responsible authorities and institutions should increase their efforts to decrease the stray dog population, and to control and apply preventative measures to limit the spread of dirofilariasis. To the best of our knowledge, our findings on *A*. *reconditum* and *D*. *repens* represent the first report of these pathogens in the canine population of the Republic of Moldova.

## Conclusions

The present epidemiological survey confirms the presence in the Republic of Moldova of *D*. *immitis*, *D*. *repens* and *A*. *reconditum* in dogs that did not receive any heartworm preventive. The data reported here extend our knowledge of the geographical distribution of these nematodes and highlight the need for the development of programmes to prevent their spread because of the deleterious effects that they can have on animal and human health.

## Data Availability

Some of the datasets generated, used and analysed during the current study are included in this published article and some of the data are available from the corresponding author on reasonable request. The four sequences generated herein were deposited in GenBank under accession numbers MW656248-MW656251.

## Abbreviations

BLAST: Basic Local Alignment Search Tool
bp: Base pair
CI: Confidence interval
PCR: Polymerase chain reaction
